# Development and internal validation of a mammography-based model fusing clinical, radiomics, and deep learning models for sentinel lymph node metastasis prediction in breast cancer

**DOI:** 10.3389/fmed.2025.1659422

**Published:** 2025-09-09

**Authors:** Xingyuan Liu, Ye Ruan, Siwei Cao, Mingming Zhao, Zhongxing Shi, Yantong Jin, Yang Wang, Bo Gao

**Affiliations:** ^1^Department of Radiology, The Second Affiliated Hospital of Harbin Medical University, Harbin, China; ^2^Department of Interventional Radiology, The Second Affiliated Hospital of Harbin Medical University, Harbin, China

**Keywords:** breast cancer, radiomics, sentinel lymph node, machine learning, full-field digital mammography, information fusion

## Abstract

**Objective:**

To develop a mammography (MG)-based post-fusion model combined with Clinical, Radiomics, and Deep Learning Models to evaluate the status of sentinel lymph node (SLN) in patients with breast cancer.

**Methods:**

A total of 290 breast cancer patients who underwent MG were randomly divided into a training set (*n* = 203) and an internal validation set (*n* = 87), with an additional 82 patients included in the test set for independent validation. From the MG images of mediolateral oblique (MLO) and craniocaudal (CC) views, 1726 radiomic (Rad) features and 1,024 deep learning (DL) features were extracted for each patient. After the feature fusion and selection, the single-modal models and pre-fusion models were established by stochastic gradient descent (SGD). Using the probabilities of single-modal models, the post-fusion models were developed by support vector machine (SVM). The area under the receiver operating characteristic curve (AUC) was used for accessing the performance of models. The clinical net benefit and predictive accuracy were evaluated through decision curve analysis (DCA) and calibration curves.

**Results:**

The post-fusion model Clinical+Rad+DL combined probabilities of single modal models, showed the best discrimination ability in the internal validation set (AUC [95%CI]: 0.845 [0.769–0.921]) and test set (AUC [95%CI]: 0.825 [0.812–0.932]).

**Conclusion:**

The proposed post-fusion model Clinical+Rad+DL demonstrated the method of probabilities fusion was effective and showed promise for predicting SLN metastasis in breast cancer.

## 1 Introduction

Breast cancer is the most commonly diagnosed cancer and the leading cause of cancer-related death in women (1). Axillary lymph node (ALN) status is critical in staging breast cancer and guiding treatment decisions (2, 3). Sentinel lymph node biopsy (SLNB) has become the preferred method for assessing ALN metastasis in early-stage breast cancer patients because SLN is recognized as the primary site for tumor spread to the axillary region (4). However, it’s important to note that SLNB is an invasive procedure that can lead to complications such as axillary wound infection, seroma formation, and paresthesias (5). That being said, ultrasound (6, 7), mammography (MG) (8, 9), and magnetic resonance imaging (MRI) (10) detect lymph node metastasis by identifying morphological and functional characteristics, but their sensitivity and specificity do not meet clinical needs.

Radiomics (Rad) is a non-invasive method that involves the high-throughput extraction of large amounts of image features from radiographic images to predict tumor diagnosis and prognosis (11). Several studies have applied Rad features to predict SLN metastasis in breast cancer (12, 13). Moreover, it is worth noting that deep learning (DL) has been widely employed in breast MRI (14–17) and breast ultrasound (18–20) for various tasks, including segmentation, diagnosis, grading, and metastasis prediction. DL features have the potential to provided more comprehensive information than Rad features, as they can capture complex and subtle features within images. The combination of Rad and DL features may potentially enhance the model’s performance. Various methods for fusion have been proposed, including feature fusion (pre-fusion) and probability fusion (post-fusion). In a study by Xie et al. (21), an approach was proposed that integrates decision-level texture, shape, and DL features for classifying lung nodules. Furthermore, Li et al. (22) utilized a probabilistic fusion technique to create a model based on MRI for forecasting ALN metastasis, which yielded an AUC of 0.91. This level of performance exceeded that of both the Rad and DL models. These studies indicate that the use of post-fusion techniques, such as probability fusion, to construct predictive models for breast cancer SLN metastasis exhibits potential.

Thus, our study aimed to develop and compare pre-fusion and post-fusion models encompassing clinical, Rad, and DL features of MG to predict SLN metastasis in breast cancer.

## 2 Materials and methods

### 2.1 Patient population

The research was conducted in accordance with the ethical guidelines established in the 2013 revision of the Declaration of Helsinki for studies involving human participants. Approval for the study was obtained from the institutional review committee of our hospital. Since the study had a retrospective design, the requirement for informed consent was exempt.

A total of 290 patients diagnosed with invasive breast carcinoma between March 2016 and June 2023 were enrolled, while 82 patients diagnosed between January 2014 and February 2016 served as an independent test set. The inclusion criteria were as follows: (1) Underwent MG examination within the 2 weeks before surgery, and the images met the diagnostic requirements; (2) Underwent SLNB during surgery to assess the status of the SLN. The exclusion criteria were as follows: (1) Had chemotherapy, radiotherapy, or endocrine therapy before surgery; (2) Received treatment or biopsy before MG examination; (3) Diagnosed with bilateral, multicentric, multifocal breast cancer, or evidence of distant metastasis. The flowchart for enrolled patients is illustrated in Supplementary Figure 1.

**Figure 1:**
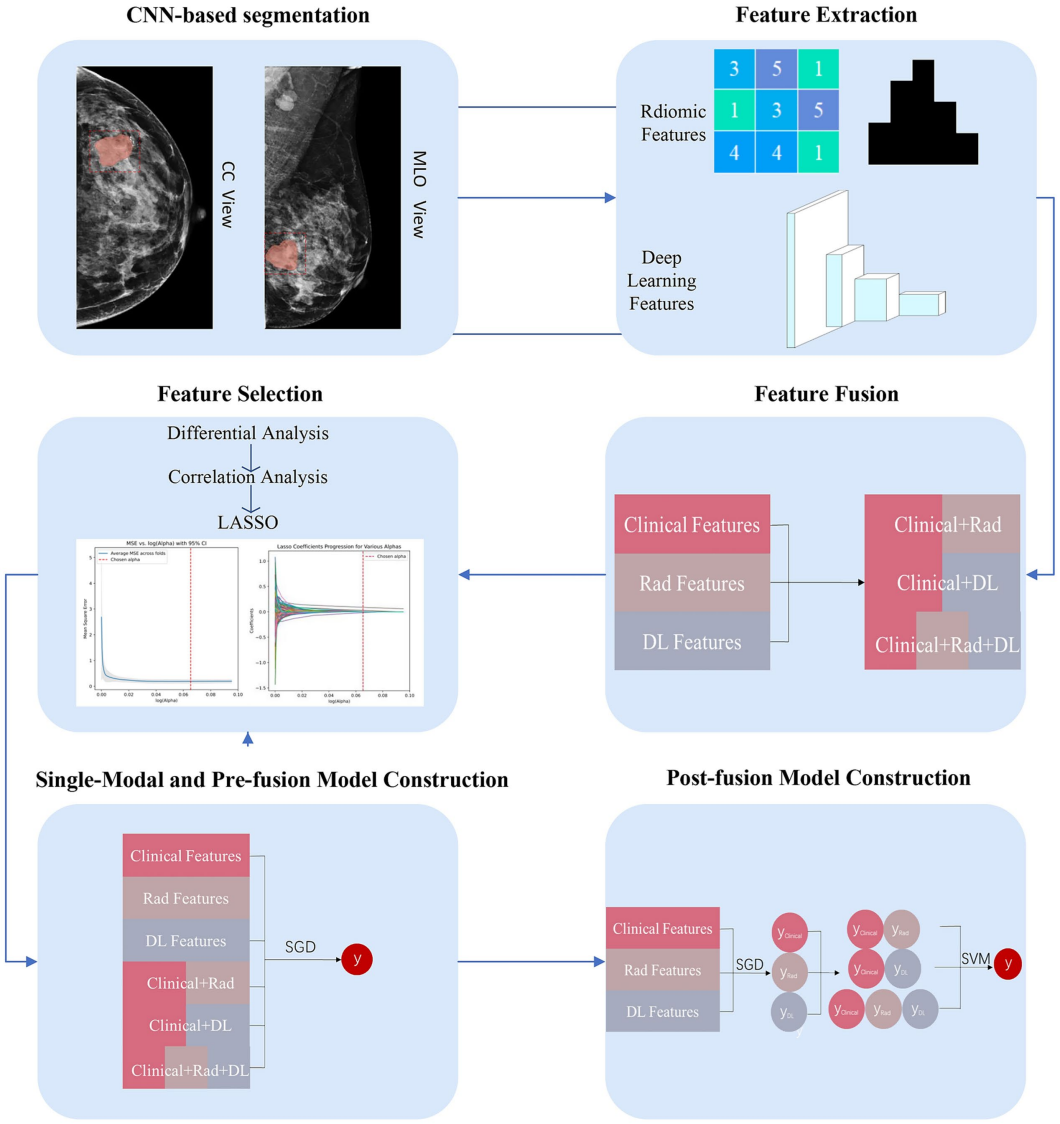
Flow chart of cohort selection.

The patient data included the following datasets: (a) A regions of interest (ROIs) training set used to train a DL segmentation model for identifying MG lesions; (b) An ROIs validation set used to assess the DL segmentation model’s performance in ROIs segmentation; (c) A radiomics dataset where patients were randomly divided into a training set and an internal validation set at a 7:3 ratio; (d) A test set from a separate cohort was included to further evaluate the model’s performance. Ultimately, 290 patients and 82 test patients were enrolled in the study. The cohort selection flowchart is shown in Figure 1.

### 2.2 MG examination and image acquisition

The Hologic Selenia full digital MG camera (Hologic Medical Systems, Boston, MA) was utilized to conduct bilateral digital MG examinations, acquiring digital MG images in mediolateral oblique (MLO) and craniocaudal (CC) views. The images were analyzed using a Hologic breast computer-aided diagnosis workstation (SecureViewDx; Hologic) equipped with two 5-megapixel monitors, each with a resolution of 1792 × 2048.

### 2.3 Assessment of conventional semantic features in MG and clinicopathologic characteristics

The evaluation of the conventional semantic features of MG was carried out by two experienced breast imaging radiologists, Radiologists 1 and Radiologists 2, who have 30 and 10 years of expertise in MG diagnosis, respectively. The assessment was conducted using the workstation without prior knowledge of the pathological outcomes. The study examined the conventional semantic features of MG based on the American College of Radiology Breast Imaging Reporting and Data System (ACR BI-RADS) 5th edition standard. This included analyzing diameter, shape (round or oval/irregular), glandular type (non-dense breast/dense breast), margin (spiculated/non-spiculated), mass density (high density/equal density), suspicious morphology of calcifications (absent/present). In addition, suspicious lymph node signs in MG included rounded or irregular shape, absence of fatty hilum, small diameter ≤1 cm, and increased density. If any of these signs were present, the MG-reported abnormal lymph node (MG_reported_LN) was recorded as positive8, 9.

The agreement of the conventional semantic features of MG was analyzed using the Kappa test.

Clinicopathologic features included the patient’s age, weight, height, body mass index (BMI), neutrophil-to-lymphocyte ratio (NLR), estrogen receptor (ER) status (positive/negative), progesterone receptor (PR) status (positive/negative), human epidermal growth factor receptor-2 (HER-2) status (positive/negative), Ki-67 (≥30%/<30%) status, and histological grading (I/II/III).

The conventional semantic features and clinicopathologic features were defined as the Clinical features.

### 2.4 CNN-based MG images segmentation

**Figure 2:**
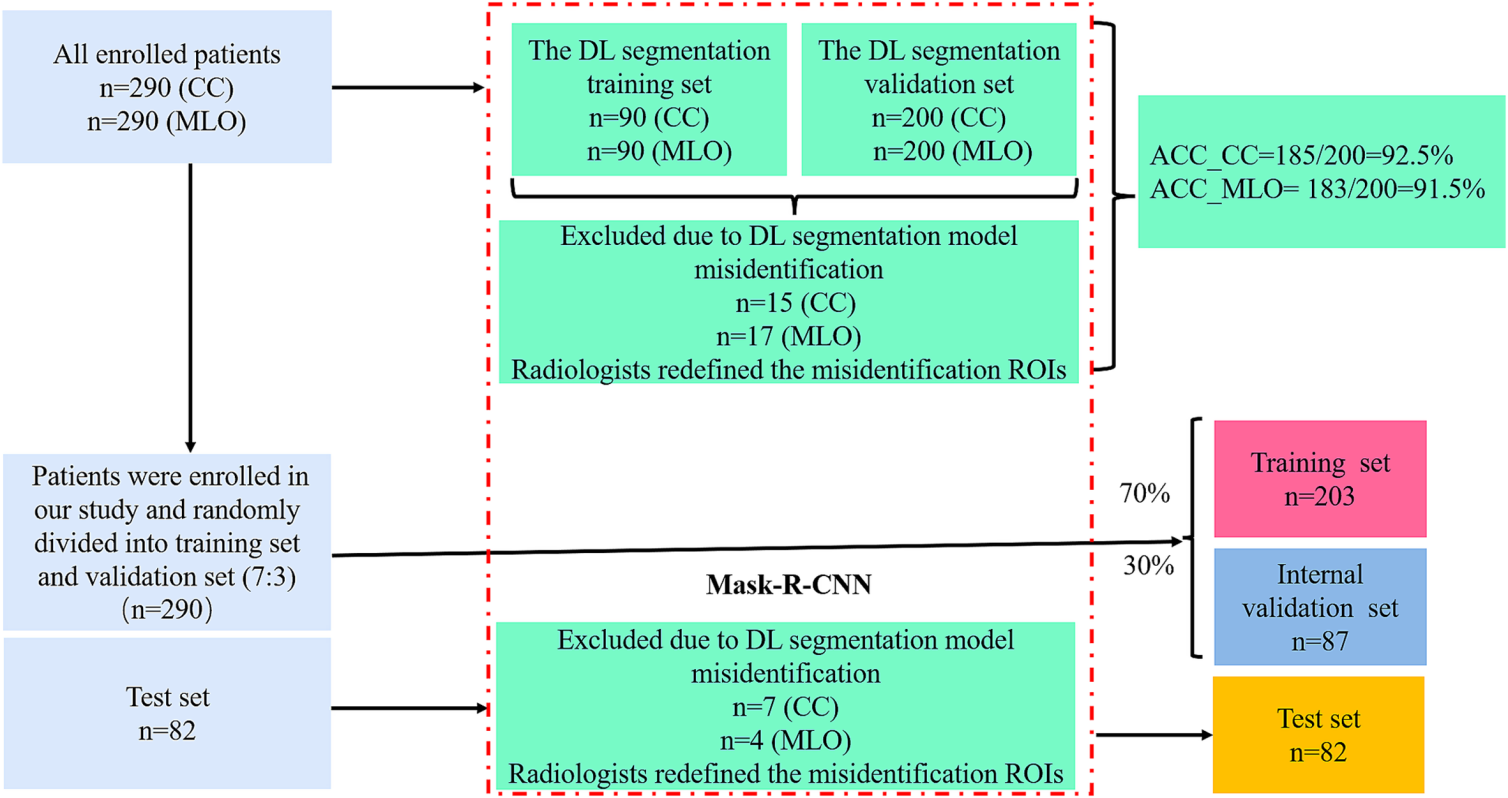
The design of workflow for the study.

The workflow is shown in Figures 1, 2. Radiologists first randomly selected 90 patients, including their MLO and CC view images, and performed manual segmentation using 3D Slicer (version 5.2.2), a tool widely used for accurate medical image annotation, to generate labeled data for the segmentation task. This labeled dataset formed the basis for training a DL segmentation model using the Mask-R-Convolutional Neural Network (Mask-R-CNN) architecture, chosen for its proven effectiveness in medical image segmentation. The convolutional layers of the Mask-R-CNN model were initially pre-trained on the Microsoft Common Objects in Context (COCO) dataset to acquire general feature representations, with a learning rate of 0.001, a batch size of 10, and a total of 100 epochs. Subsequently, the model was fine-tuned using the DL segmentation training set and validated on the DL segmentation validation set to evaluate and optimize its segmentation performance. Finally, images excluded due to DL segmentation model misidentification (e.g., failure to identify tumor boundaries or incorrect ROIs placement) were re-annotated with ROIs by Radiologists. The corrected data were then integrated back into datasets.

### 2.5 Radiomic feature extraction and DL feature extraction

Feature extraction was carried out using the open-source software PyRadiomics (version 3.1.0). A total of 1726 Rad features were extracted from two regions of interest (CC and MLO views) for each patient. These features included shape, intensity, textural, and wavelet features.

In recent years, ResNet has been shown to have excellent performance and good applications in medical imaging tasks (23, 24). We adopted a pre-trained ResNet18 model by maintaining the original kernel size, stride, and padding settings, allowing for direct application of deep learning feature extraction from medical images. We used SimpleITK (version 2.2.0) to read images and ROIs and convert them into Numpy arrays. We then normalized and standardized these arrays. To make the model output features rather than classification results, we removed the last full connection layer of the model to obtain the intermediate features from the penultimate layer. In total, 1,024 features were extracted from 2 ROIs (CC and MLO views) for each patient. All features were normalized using the z-score method, converting them to a standardized range of values.

### 2.6 Feature fusion and selection

To construct the pre-fusion model, we combined three types of features (Clinical, Rad, DL features) separately to get 3 types of pre-fusion features (Clinical+Rad, Clinical+DL, Clinical+Rad+DL) (Figure 2).

To obtain the features most closely associated with SLN metastasis in the training set, a three-step selection process was performed. First, we used differential analysis (Mann–Whitney U-test or independent t-test was performed for quantitative features, while chi-squared test or Fisher’s exact test was applied for categorical features) with a *p*-value threshold of 0.05 to obtain the features associated with SLN status. Then, taking into account the correlations between features, we calculated the correlation coefficients between features using Pearson or Spearman correlation. If the correlation coefficient between two features exceeded 0.75, one of the features was eliminated. Finally, the Least Absolute Shrinkage and Selection Operator (LASSO) with fivefold cross-validation to tune the parameters with the minimum lambda was used to select the optimal features.

### 2.7 Models’ development

In our study, we developed three types of models using the selected features: 1. Single-modal models (Clinical model, Rad model, and DL model); 2. Pre-fusion models: These models used the fusion features to construct an integrated model (pre-fusion model Clinical+Rad, pre-fusion model Clinical+DL, and pre-fusion model Clinical+Rad+DL); 3. Post-fusion models: these models integrated probabilities from meta-classifiers constructed separately on single-modal models to build an integrated model (post-fusion model Clinical+Rad, post-fusion model Clinical+DL, and post-fusion model Clinical+Rad+DL). The stochastic gradient descent (SGD) was utilized to construct single-modal models and pre-fusion models, and the support vector machine (SVM) was employed to develop the post-fusion models. These machine-learning algorithms have been proven to have good applications in the medical field (25, 26).

The AUC of the receiver operating characteristic (ROC) curves, accuracy, sensitivity, and specificity were used to evaluate the performance of the models. The AUCs were compared using the Delong test. Calibration curves were performed to evaluate the goodness of fit of the models. In addition, the clinical benefits of the models were assessed using the decision curve analysis (DCA) (27).

### 2.8 Statistical analysis

All statistical work, feature extraction of Rad and DL as well as model construction were conducted in Python (version 3.12.3),^1^ along with open source packages such as PyTorch, Scipy, and scikit-learn. Quantitative features were presented as means with standard deviations or as medians with the 25th and 75th percentiles. The independent sample t-test or the Mann–Whitney U-test was used for analyzing the quantitative features, while the chi-square test or the Fisher’s exact test was used for analyzing the categorical features. All tests were two-sided, with *p*-values <0.05 considered statistically significant.

## 3 Results

### 3.1 Clinical features

The clinical features of the patients are shown in Table 1. In both the training, internal validation and test sets, margin, MG_reported_LN showed the most significant differences in distribution between the SLN+ and SLN− groups (*p* < 0.05), indicating that these three features have certain differences in their ability to predict SLNM. Although no differences were observed in the internal validation set, shape and diameter showed statistically significant differences in the training set. In addition, no statistical differences were found for NLR, ER status, PR status, HER-2 status, Ki-67 and other clinical features.

**Table 1:** Clinical features of the patients.

Clinical features	Training set (*n* = 203)			Internal validation set (*n* = 87)			Test set (*n* = 82)		
	SLN− (*N* = 147)	SLN+ (*N* = 56)	*p*-value	SLN− (*N* = 63)	SLN+ (*N* = 24)	*p*-value	SLN− (*N* = 59)	SLN+ (*N* = 23)	*p*-value
Age	56 (46, 64)	54 (46, 60)	0.47	53 (42, 60)	57 (46, 64)	0.24	57 (46, 65)	54 (45, 61)	0.34
Diameter	2.00 (1.70, 2.50)	2.35 (1.90, 3.00)	0.009	2.10 (1.85, 2.50)	2.10 (1.80, 2.40)	0.94	2.10 (1.73, 2.50)	2.10 (1.78, 2.63)	0.49
Weight	61 (57, 70)	60 (55, 70)	0.69	60 (55, 65)	62 (55, 70)	0.25	60 (57, 69)	62 (57, 69)	0.76
Height	1.60 (1.58, 1.65)	1.60 (1.58, 1.62)	0.31	1.60 (1.58, 1.62)	1.60 (1.59, 1.64)	0.51	1.60 (1.60, 1.66)	1.60 (1.59, 1.63)	0.16
BMI	23.8 (22.0, 26.6)	23.6 (21.7, 26.6)	0.79	23.4 (22.03, 24.99)	24.1 (22.48, 26.03)	0.23	23.44 (21.49, 25.24)	23.53 (22.53, 26.73)	0.28
NLR	1.82 (1.42, 2.27)	1.83 (1.46, 2.27)	0.74	1.74 (1.31, 2.36)	1.82 (1.52, 2.33)	0.73	1.87 (1.43 2.37)	1.82 (1.47, 2.13)	0.76
Breast Composition			0.9			0.91			0.88
Non-dense breast	75 (51%)	28 (50%)		35 (56%)	13 (54%)		28 (48.3%)	12(50.0%)	
Dense breast	72 (49%)	28 (50%)		28 (44%)	11 (46%)		30 (51.7%)	12(50.0%)	
Density			0.3			0.91			0.18
Equal density	88 (60%)	29 (52%)		35 (56%)	13 (54%)		36 (62.1%)	11(45.8%)	
High density	59 (40%)	27 (48%)		28 (44%)	11 (46%)		22(37.9%)	13 (54.2%)	
Shape			0.02			0.54			0.14
Round or Oval	42 (29%)	7 (13%)		13 (21%)	3 (13%)		19 (32.8%)	4 (16.7%)	
Irregular	105 (71%)	49 (88%)		50 (79%)	21 (88%)		39 (67.2%)	20 (83.3%)	
Margin			<0.001			0.03			0.03
Non-spiculated	37 (25%)	36 (64%)		21 (33%)	14 (58%)		37 (63.8%)	9 (37.5%)	
Spiculated	110 (75%)	20 (36%)		42 (67%)	10 (42%)		21 (36.2%)	15 (62.5%)	
Calcifications			0.04			0.71			0.05
Absent	94 (64%)	27 (48%)		42 (67%)	15 (63%)		42 (72.4%)	12 (50.0%)	
Present	53 (36%)	29 (52%)		21 (33%)	9 (38%)		16 (27.6%)	12 (50.0%)	
MG_reported_LN			<0.001			<0.001			0.02
Negative	118 (80%)	24 (43%)		55 (87%)	12 (50%)		52 (89.7%)	16 (66.7%)	
Positive	29 (20%)	32 (57%)		8 (13%)	12 (50%)		6 (10.3%)	20 (83.3%)	
ER			0.5			0.02			0.77
Negative	23 (16%)	11 (20%)		17 (27%)	1 (4%)		12 (20.7%)	4 (16.7%)	
Positive	124 (84%)	45 (80%)		46 (73%)	23 (96%)		46 (79.3%)	170 (81%)	
PR			0.55			0.009			0.89
Negative	36 (24%)	16 (29%)		23 (37%)	2 (8%)		16 (27.6%)	7 (29.2%)	
Positive	111 (76%)	40 (71%)		40 (63%)	22 (92%)		42 (72.4%)	17 (70.8%)	
HER-2			0.66			0.16			0.12
Negative	122 (83%)	45 (80%)		51 (81%)	16 (67%)		50 (86.2%)	17 (70.8%)	
Positive	25 (17%)	11 (20%)		12 (19%)	8 (33%)		8 (13.8%)	7 (29.2%)	
KI-67			0.98			0.68			0.95
<0.3	79 (54%)	30 (54%)		31 (49%)	13 (54%)		31 (53.4%)	13 (54.2%)	
≥0.3	68 (46%)	26 (46%)		32 (51%)	11 (46%)		27 (46.6%)	11 (45.8%)	
Histological Grading			0.43			0.69			0.98
I	19 (13%)	4 (7%)		4 (6%)	2 (8%)		6 (10.3%)	2 (8.3%)	
II	73 (50%)	32 (57%)		37 (59%)	12 (50%)		33 (56.9%)	14 (58.3%)	
III	55 (37%)	20 (36%)		22 (35%)	10 (42%)		19 (32.8%)	8 (33.3%)	

ER, estrogen receptor; HER-2, human epidermal growth factor receptor-2; NLR, neutrophil-to-lymphocyte ratio; PR, progesterone receptor; SLN+, sentinel lymph node with metastasis; SLN−, sentinel lymph node without metastasis.

### 3.2 MG images segmentation and feature selection

185 CC views and 183 MLO views from 400 images of 200 patients were accurately segmented, with an accuracy of 92.5% for the CC set and 91.5% for the MLO set, respectively.

The kappa values for conventional semantic features of MG by two radiologists were all >0.80.

We implemented an independent feature selection approach for each feature set within the training set. After feature selection, the features of the single-modal models and the pre-fusion models are shown in Supplementary Table 1. For the Clinical model in the single-modal models, the features diameter, shape, margin, and MG_reported_LN and were selected.

### 3.3 Model construction and performance

**Figure 3:**
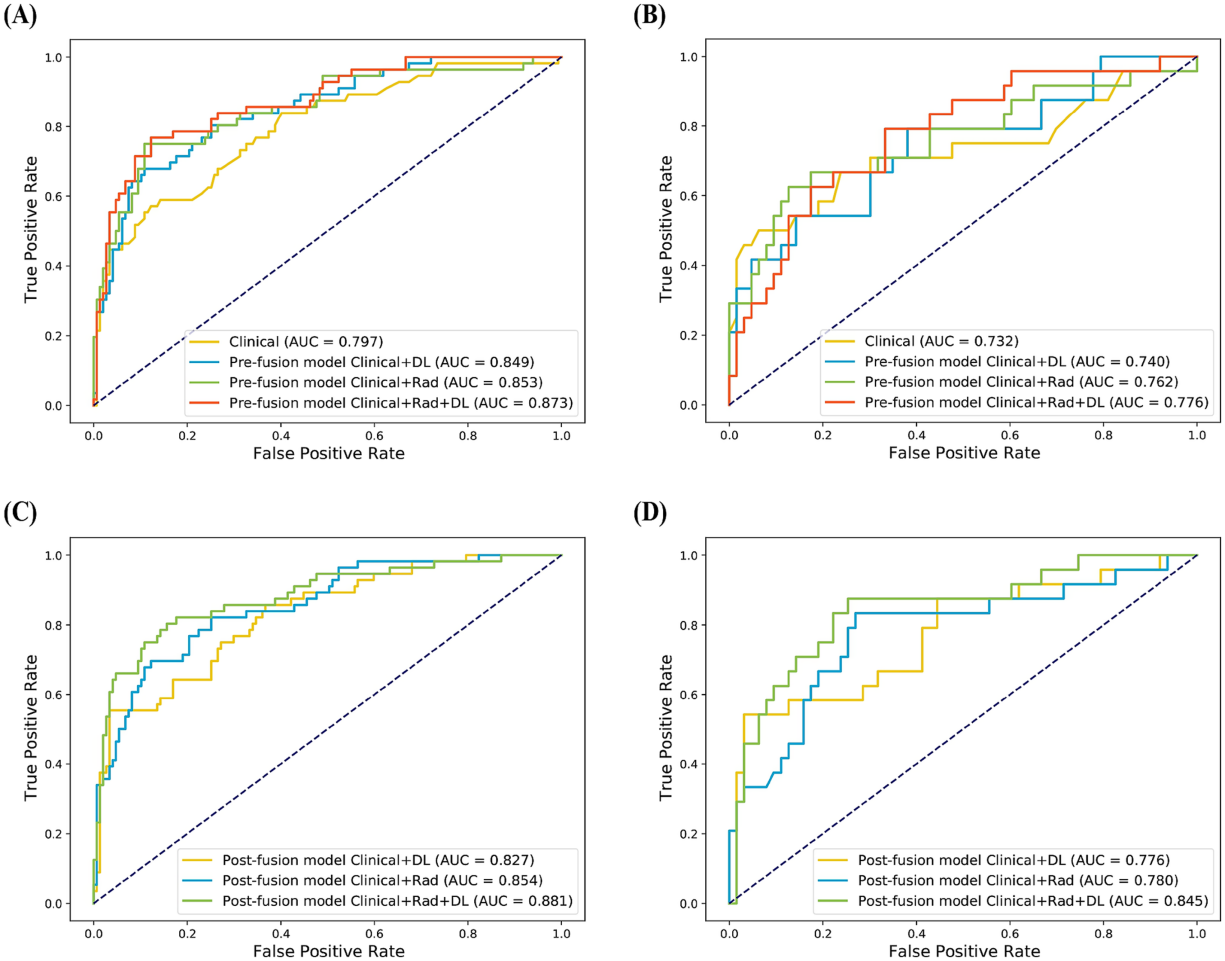
Receiver operating characteristic (ROC) curves of the Clinical model and pre-fusion models in the training **(A)** and validation set **(B)**. ROC curves of the post-fusion models in the training **(C)** and validation set **(D)**.

Three single-modal models were built based on the selected features. The AUC of these models (Clinical model, Rad model, and DL model) were 0.797, 0.834, and 0.744 in the training set and 0.732, 0.793 and 0.726 in the internal validation set (Table 2; Supplementary Table 2). Second, the selected pre-fusion features were used to build the pre-fusion models: Clinical+Rad, Clinical+DL, and Clinical+Rad+DL. Among these pre-fusion models, the Clinical+Rad+DL model achieved the best performance with an AUC, accuracy, sensitivity and specificity of 0.873, 0.847, 0.768 and 0.878, respectively, in the training set and 0.776, 0.701, 0.791 and 0.667, respectively, in the internal validation set (Table 2; Figure 3). Finally, the prediction probabilities of the three single-modal models were further fused using SVM to build post-fusion models. The prediction probabilities of the Clinical model and the Rad model were fused to construct the post-fusion model Clinical+Rad; the prediction probabilities of the Clinical model and the DL model were combined to construct the post-fusion model Clinical+DL; and the prediction probabilities of the Clinical model, the Rad model, and the DL model were integrated to develop the post-fusion model Clinical+Rad+DL.

**Table 2:** Performance of the different models in training set and validation set.

Cohort	Model	AUC (95%CI)	Accuracy	Sensitivity	Specificity
Training set	Clinical model	0.797 (0.741–0.852)	0.793	0.571	0.878
	Pre-fusion model				
	Clinical+Rad	0.853 (0.805–0.902)	0.852	0.75	0.891
	Clinical+DL	0.849 (0.800–0.898)	0.833	0.679	0.891
	Clinical+Rad+DL	0.873 (0.827–0.919)	0.847	0.768	0.878
	Post-fusion model				
	Clinical+Rad	0.854 (0.806–0.903)	0.828	0.696	0.878
	Clinical+DL	0.827 (0.774–0.879)	0.852	0.554	0.966
	Clinical+Rad+DL	0.881 (0.836–0.925)	0.833	0.804	0.844
Internal validation set	Clinical model	0.732 (0.639–0.825)	0.816	0.5	0.937
	Pre-fusion model				
	Clinical+Rad	0.762 (0.672–0.851)	0.805	0.625	0.873
	Clinical+DL	0.74 (0.648–0.832)	0.667	0.792	0.619
	Clinical+Rad+DL	0.776 (0.688–0.863)	0.701	0.791	0.667
	Post-fusion model				
	Clinical+Rad	0.78 (0.693–0.867)	0.759	0.833	0.73
	Clinical+DL	0.776 (0.688–0.863)	0.851	0.542	0.968
	Clinical+Rad+DL	0.845 (0.769–0.921)	0.782	0.875	0.746
Test set	Post-fusion model				
	Clinical+Rad+DL	0.825 (0.812–0.932)	0.862	0.779	0.883

AUC, Area under the curve; DL, deep learning; Rad, radiomics.

The post-fusion model Clinical+Rad+DL showed the best performance among all models (Table 2; Figure 3). Table 3 shows the DeLong test results comparing the Clinical model, the pre-fusion model Clinical+Rad+DL, and the post-fusion model Clinical+Rad+DL. In the training set, this model achieved the highest AUC of 0.881, which was statistically significantly higher than both the Clinical model (*p* < 0.001) and the pre-fusion model Clinical+Rad+DL (*p* = 0.03). Similarly, in the internal validation set, the AUC (0.845) of the post-fusion Clinical+Rad+DL model was the highest and statistically significant when compared to the Clinical model (*p* = 0.04) and the pre-fusion Clinical+Rad+DL model (*p* = 0.04).

**Table 3:** Comparison of diagnostic performance between different models.

Model vs model	*p*-value
Training set	
Clinical vs. Pre-fusion Clinical+Rad+DL	0.032
Clinical vs. Post-fusion Clinical+Rad+DL	0.044
Pre-fusion Clinical+Rad+DL vs. Post-fusion Clinical+Rad+DL	0.03
Internal validation set	
Clinical vs. Pre-fusion Clinical+Rad+DL	0.78
Clinical vs. Post-fusion Clinical+Rad+DL	0.038
Pre-fusion Clinical+Rad+DL vs. Post-fusion Clinical+Rad+DL	0.027

DL, deep learning; Rad, radiomics.

**Figure 4:**
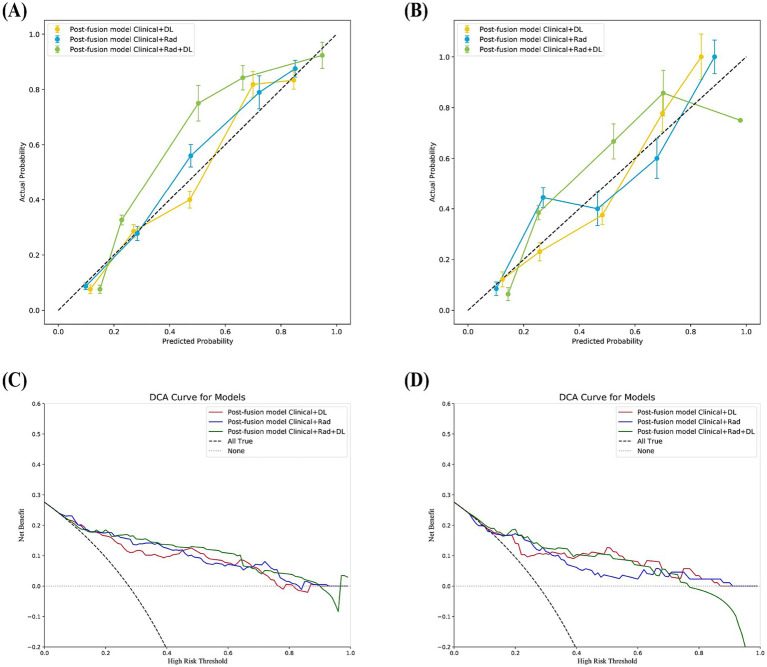
The calibration curves of post-fusion models in the training **(A)** and validation set **(B)**. Calibration curves demonstrate the goodness-of-fit of models. Decision curves analysis (DCA) for post-fusion models are showed in the training **(C)** and validation set**(D)**; the y-axis indicates the net benefit, the x-axis indicates threshold probability.

The calibration curves (Figure 4) indicated that the true statement of SLN was consistent with the result of the post-fusion model Clinical+Rad+DL in the training and internal validation sets. The DCA for the post-fusion models is shown in Figure 4. When an individual’s threshold probability is <0.77, the post-fusion model Clinical+Rad+DL would add net benefit compared to the treat-all or treat-none tactics. The calibration curves, and DCA curves of the other models are shown in Supplementary Figure 4.

**Figure 5:**
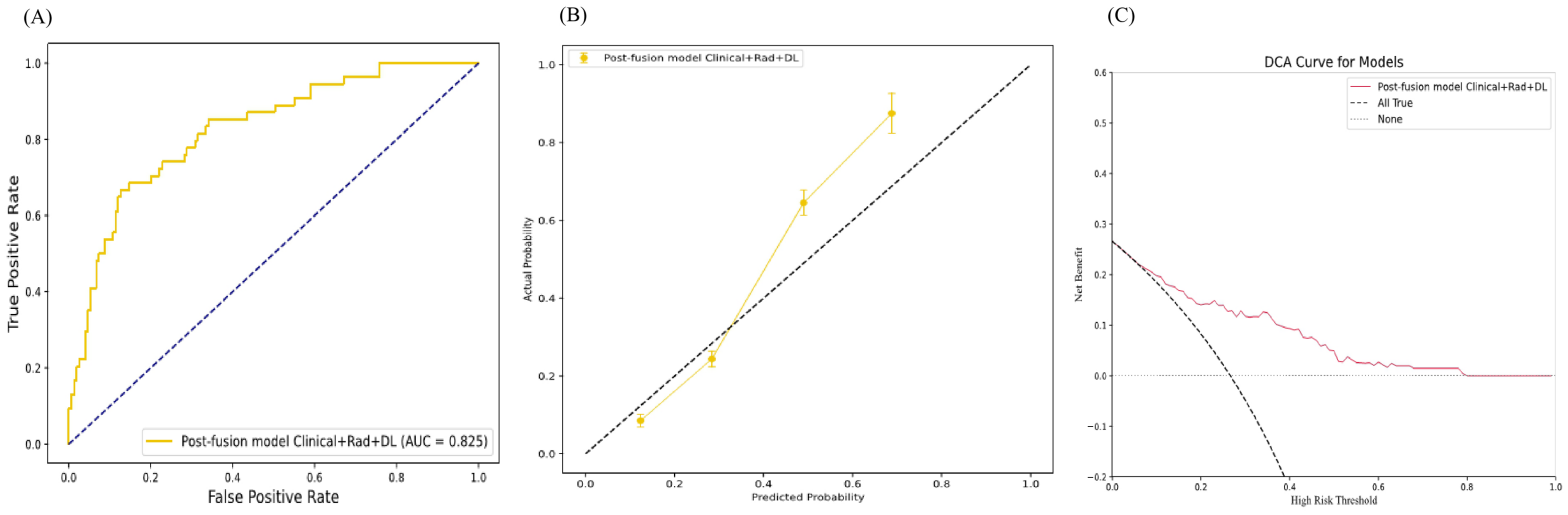
The test set performance of the Post-Fusion Model Clinical+Rad+DL. **(A)** Receiver operating characteristic (ROC) curves. **(B)** Calibration curves. **(C)** Decision curves analysis (DCA).

Finally, we applied the most optimal model, the post-fusion model Clinical+Rad+DL, to the test set, which also demonstrated good discrimination (AUC = 0.825), calibration, and clinical applicability (Figure 5).

## 4 Discussion

In this study, the post-fusion model Clinical+Rad+DL, which integrated the probabilities of the Clinical, Rad, and DL models, achieved the best performance in distinguishing SLN metastasis status. Our results indicated that the post-fusion model Clinical+Rad+DL demonstrated promising predictive performance, with important implications for surgical planning in breast cancer patients.

After feature selection, clinical features such as diameter, shape, margin, and MG_reported_LN were incorporated into the Clinical model. Many previous studies have confirmed their association with lymph node metastasis. Lyu et al. (28) found that tumor size is an independent risk factor for SLN metastasis in breast cancer. In the study by Yuan et al. (29), patients with spiculated margins on MG images were more likely to have SLN metastasis. Breast cancer shape on MG showed no statistical difference in the validation set, likely due to the small sample size and the division between training and validation sets. Although there is no literature suggesting that irregularly shaped breast cancers are more prone to lymph node metastasis on MG, breast cancers with irregular shapes on ultrasound (30) are more likely to undergo lymph node metastasis.

This Clinical model incorporating the MG_reported_LN feature showed lower sensitivity (0.5), with some studies also confirming the drawback of MG for accessing lymph node status29. One possible reason is that some patients’ axillae may not be fully exposed in the standard positions (CC and MLO views). In our study, the use of a model with the post-fusion mode Clinical+Rad+DL can compensate for this drawback (sensitivity:0.875) and also avoid errors arising from radiologists’ subjectivity and reliance on experience. Previous research on the prediction of lymph node metastasis by radiomics has mainly focused on the characteristics of the primary tumor (30, 31). Lymph node metastasis in breast cancer is a complex process, typically associated with changes in the immune microenvironment of the primary tumor region (32). Rad features have been shown to reflect the heterogeneity of the primary tumor site and the degree of immune cell infiltration (33, 34). Consequently, models based on features extracted from the primary tumor may improve model performance and serve as one of the strategies to overcome the limitations of MG.

Previous studies have shown that traditional Rad research based on MG shows promising results, with AUCs ranging from 0.767 to 0.87635-37. Compared to these previous studies, we further integrated features from ResNet18, either through pre- or post-fusion models, both of which yielded satisfactory results and demonstrated certain advantages (Table 2) in predicting SLN metastasis. In contrast to the quantified features of Rad features, DL models can extract more abstract and higher-dimensional information from images. Combining DL features with Rad features allows the complementary integration of information from both sources, enabling a more comprehensive analysis of images and thus improving the predictive ability.

In the current study, the performance of single-modal models was unsatisfactory. However, the post-fusion models using probabilistic fusion outperformed the pre-fusion models using feature fusion. Specifically, the post-fusion model combining Clinical+Rad+DL had a higher AUCs with values of 0.881 on the training set and 0.845 on the validation set. Such models using the post-fusion strategy of probabilistic fusion will perform better than the pre-fusion model, and the same conclusion has been reached in other studies (22, 35). The post-fusion model offers several advantages. First, since different models may excel in different aspects, model fusion can leverage the strengths of different models to achieve more accurate prediction results. Second, combining multiple models can mitigate the risk of overfitting associated with individual models, thereby improving the robustness and stability of the model. In addition, multi-model fusion can improve the generalization ability of the model by reducing its variance, leading to better performance on test data.

Our study has several limitations. First, it was a retrospective analysis with data collected from a single center and a relatively small sample size, and it lacked an independent external dataset for validation, which may introduce selection bias and limit the generalizability of the findings. To address this issue, future work should involve larger patient cohorts and multicenter prospective studies, which would help validate our results and enhance the robustness and clinical utility of the proposed model. Moreover, our patient cohort was heterogeneous, including different pathological subtypes and clinical stages. A more precise selection of patient subgroups may yield better predictive performance and should be further explored in future studies. Finally, our study was based solely on Rad features derived from MG images. Beyond Rad, genomics can provide rich complementary information for the diagnosis, classification, and prognosis of breast cancer (36–39). Future research should focus on integrating genomics with Rad. Genomics can provide complementary biological information to improve the interpretability of Rad features, while combining the two to construct multi-omics models may further enhance diagnostic performance and facilitate more precise breast cancer management.

## 5 Conclusion

In this study, the proposed post-fusion model Clinical+Rad+DL gets the best performance, which may be potential and perspective for patients with breast cancer to avoid ALN dissection.

## Data Availability

The datasets presented in this article are not readily available because protection of patient privacy. Requests to access the datasets should be directed to Bo Gao, gaobo72519@hrbmu.edu.cn.

## Footnotes

^1^ https://www.python.org
