# Supplementary figures and images for: Development and internal validation of a mammography-based model fusing clinical, radiomics, and deep learning models for sentinel lymph node metastasis prediction in breast cancer
Source: Front Med (Lausanne). 2025 Sep 9;12:1659422. doi: 10.3389/fmed.2025.1659422 (PMC12454431; doi:10.3389/fmed.2025.1659422)

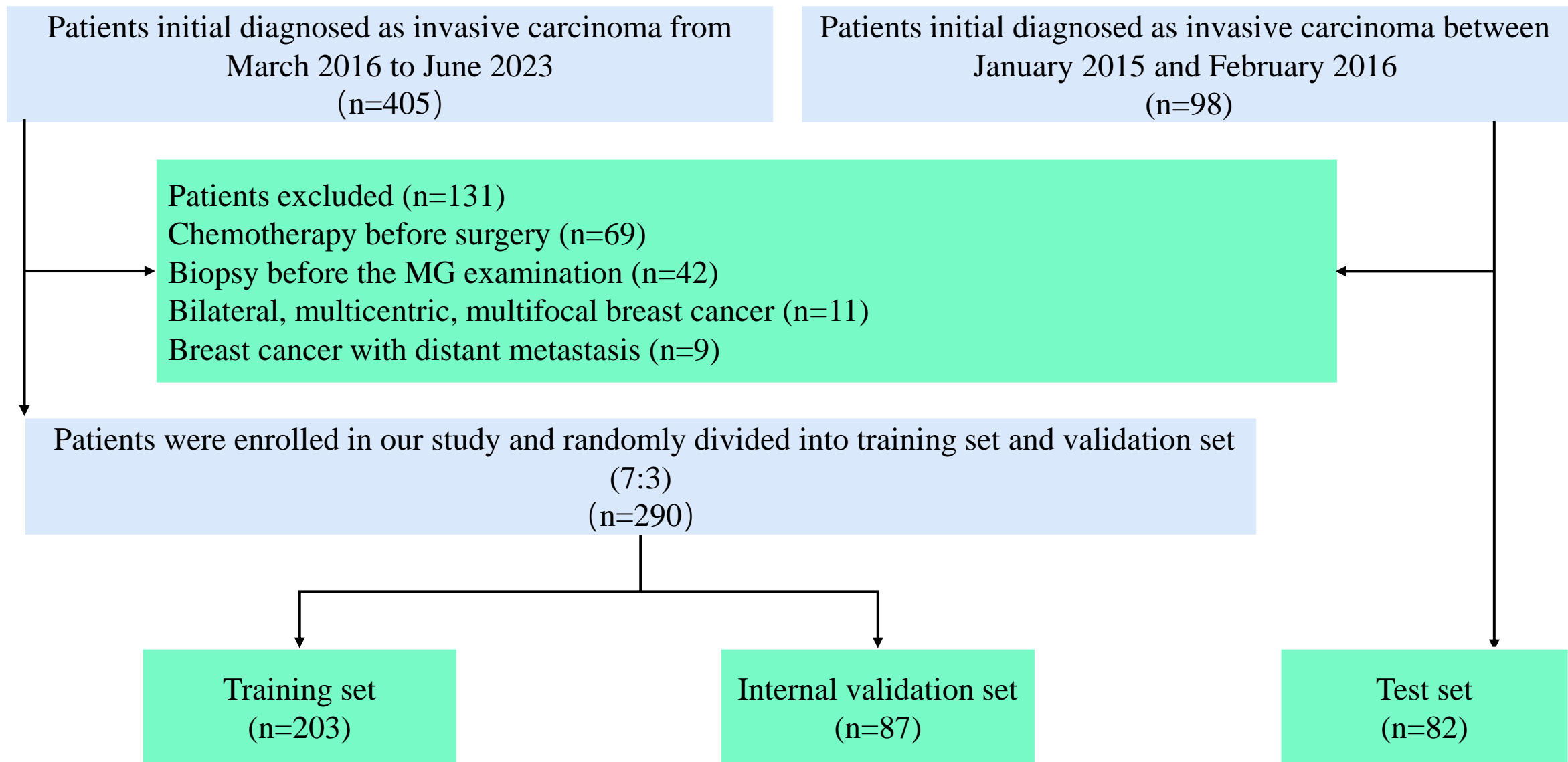

Supplement: Supplementary file 1 [file Data_Sheet_1.PDF]
