# Supplementary material for: Development and internal validation of a mammography-based model fusing clinical, radiomics, and deep learning models for sentinel lymph node metastasis prediction in breast cancer
Source: Front Med (Lausanne). 2025 Sep 9;12:1659422. doi: 10.3389/fmed.2025.1659422 (PMC12454431; doi:10.3389/fmed.2025.1659422)
Supplement: Supplementary file 2 [file Supplementary_file_1.docx]

Supplementary Figure 2 Features selection using the least absolute shrinkage and selection operator (LASSO) regression for single-modal models. Red lines are plotted at the minimum criteria. The five-fold cross-validation method was used in LASSO to screen the feature sets with the best performance chosen for Clinical model (A), Rad model (C) and DL model (E). LASSO coefficient profiles for selected features at optimal λ values, which result in non-zero coefficients for the features of Clinical model (B), Rad model (D) and DL model (F), respectively.


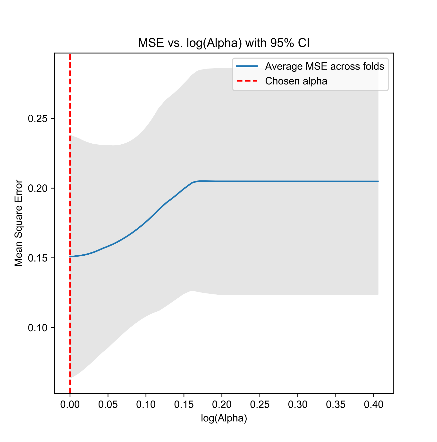

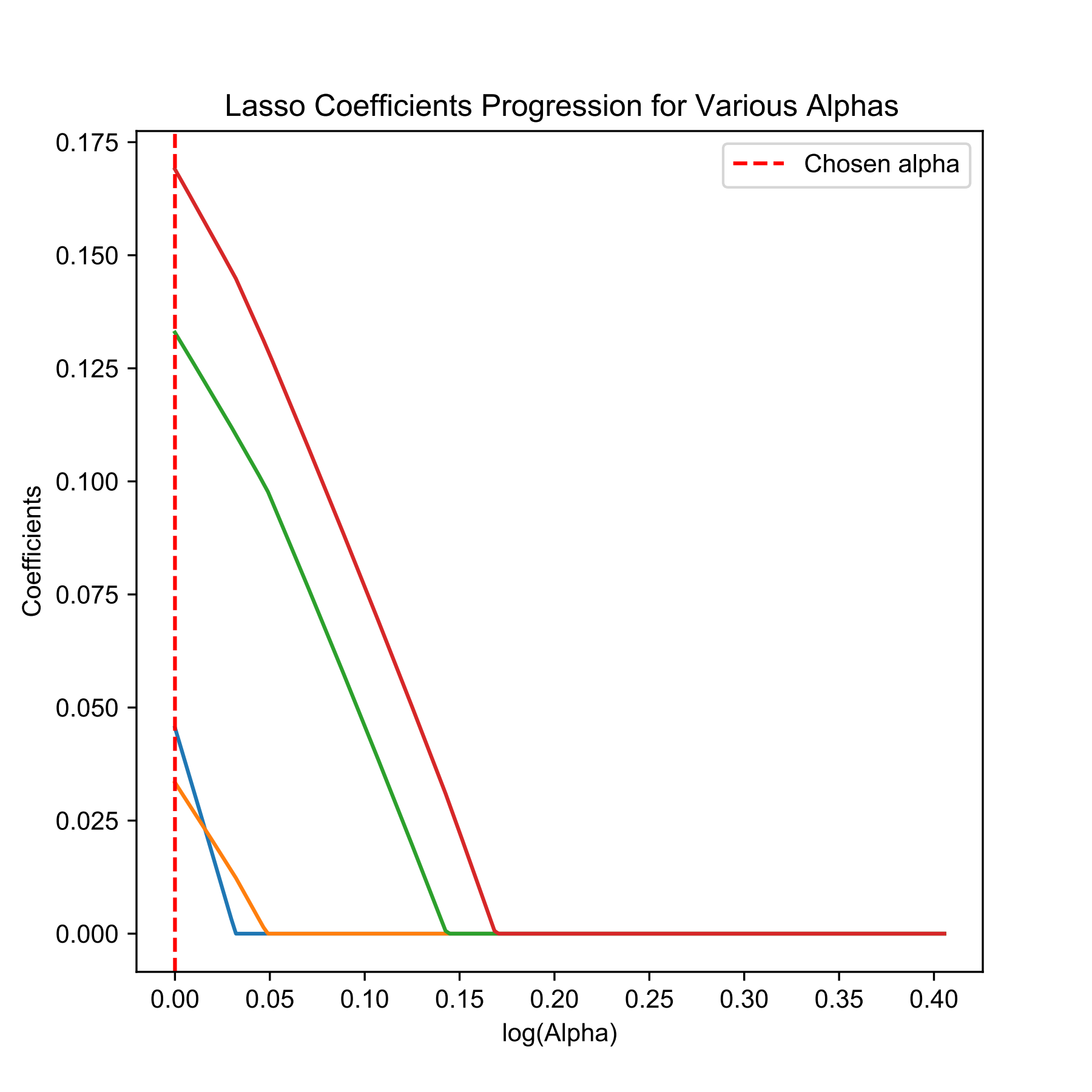

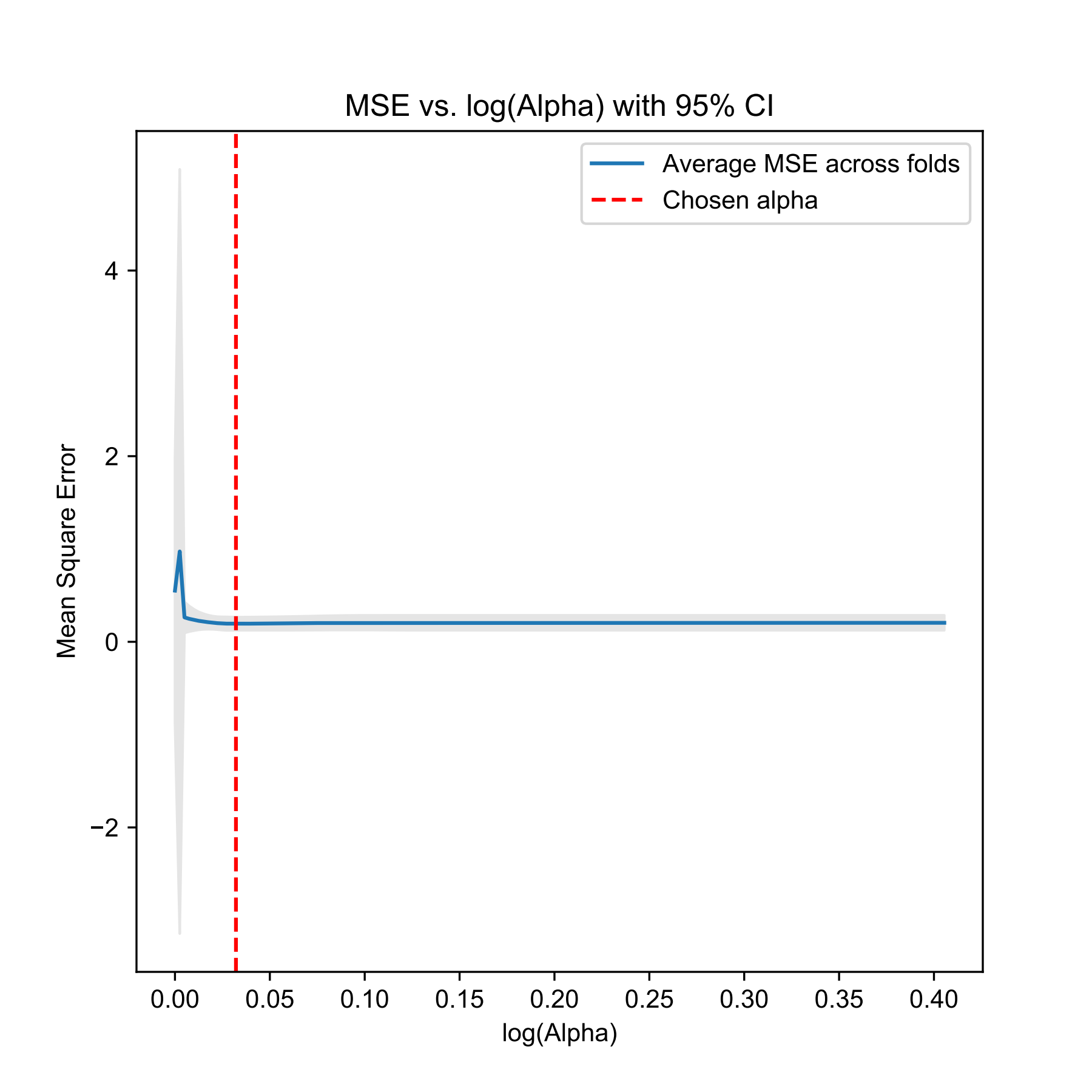

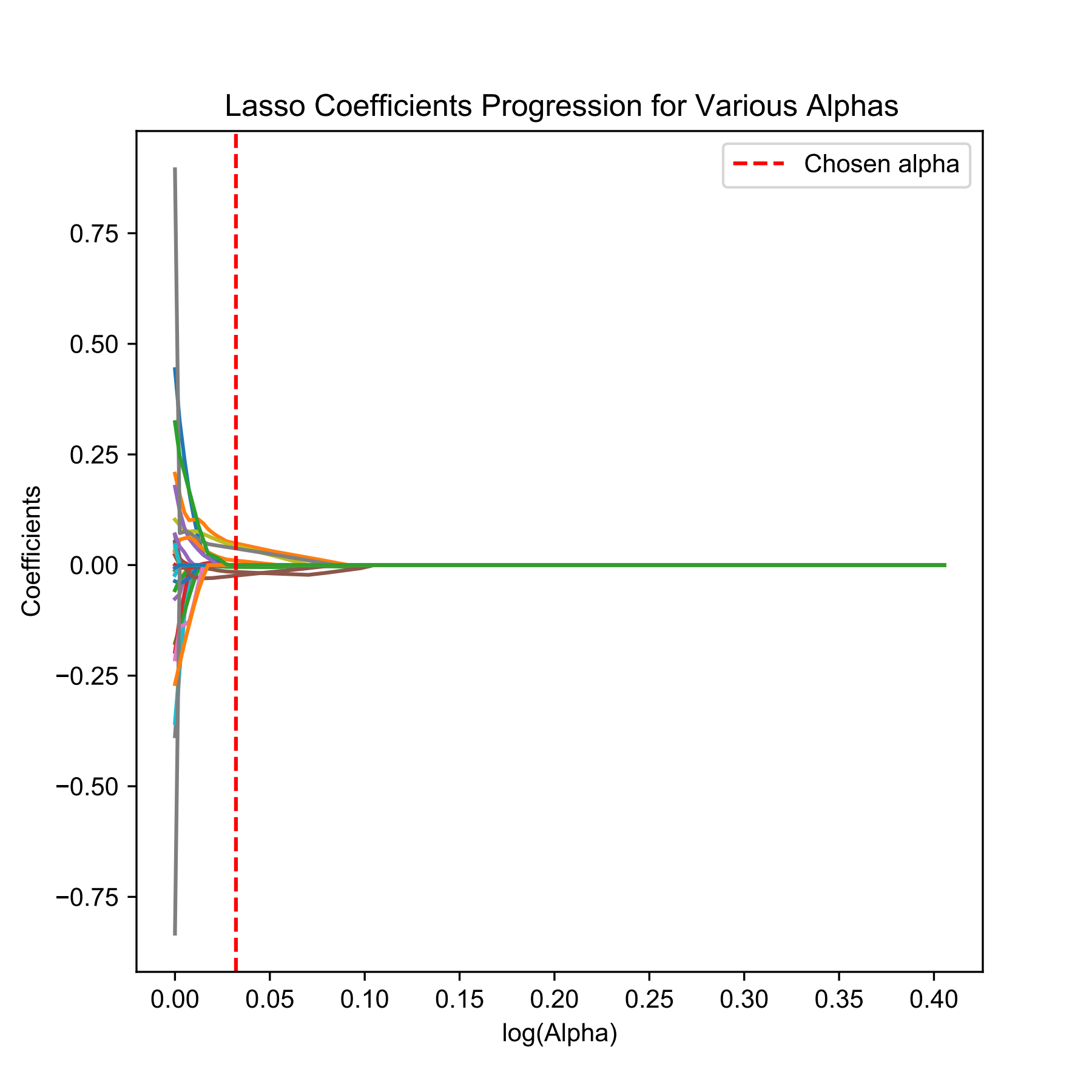


(A)

(B)


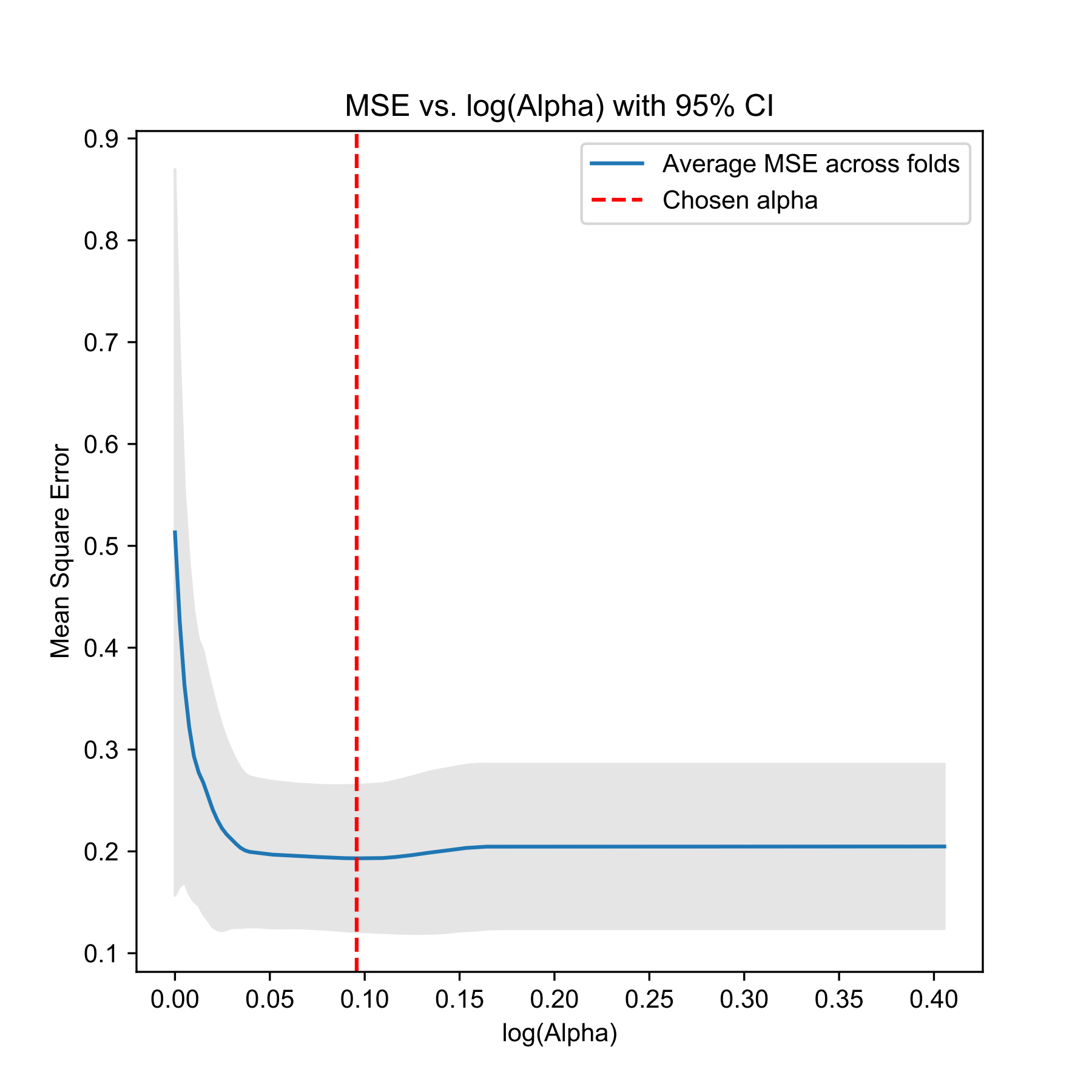

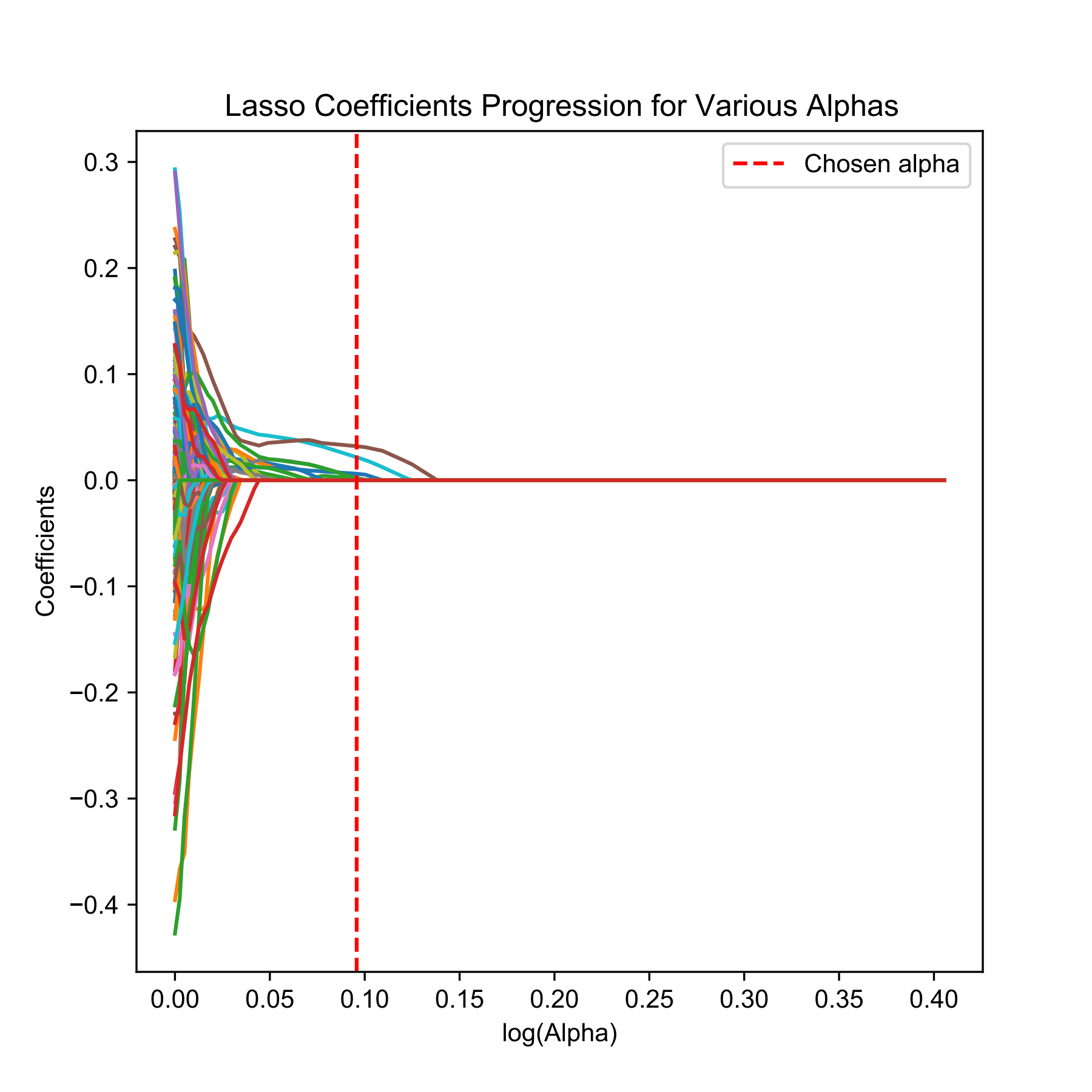


(C)

(E)

(D)

(F)

Supplementary Figure 3 Features selection using LASSO regression for pre-fusion models. Red lines are plotted at the minimum criteria. The five-fold cross-validation method was used in LASSO to screen the feature sets with the best performance chosen for pre-fusion model Clinical+Rad (A), pre-fusion model Clinical+DL (C) and pre-fusion model Clinical+Rad+DL (E). LASSO coefficient profiles for selected features at optimal λ values, which result in non-zero coefficients for the features of pre-fusion model Clinical+Rad (B), pre-fusion model Clinical+DL (D) and pre-fusion model Clinical+Rad+DL (F), respectively.


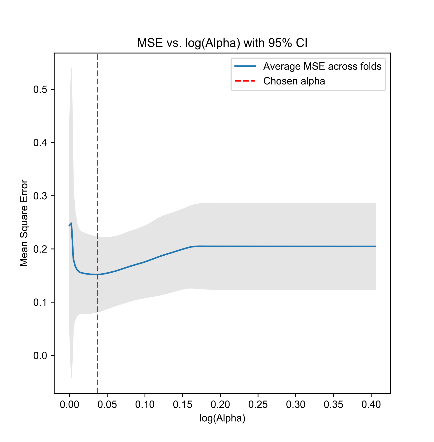

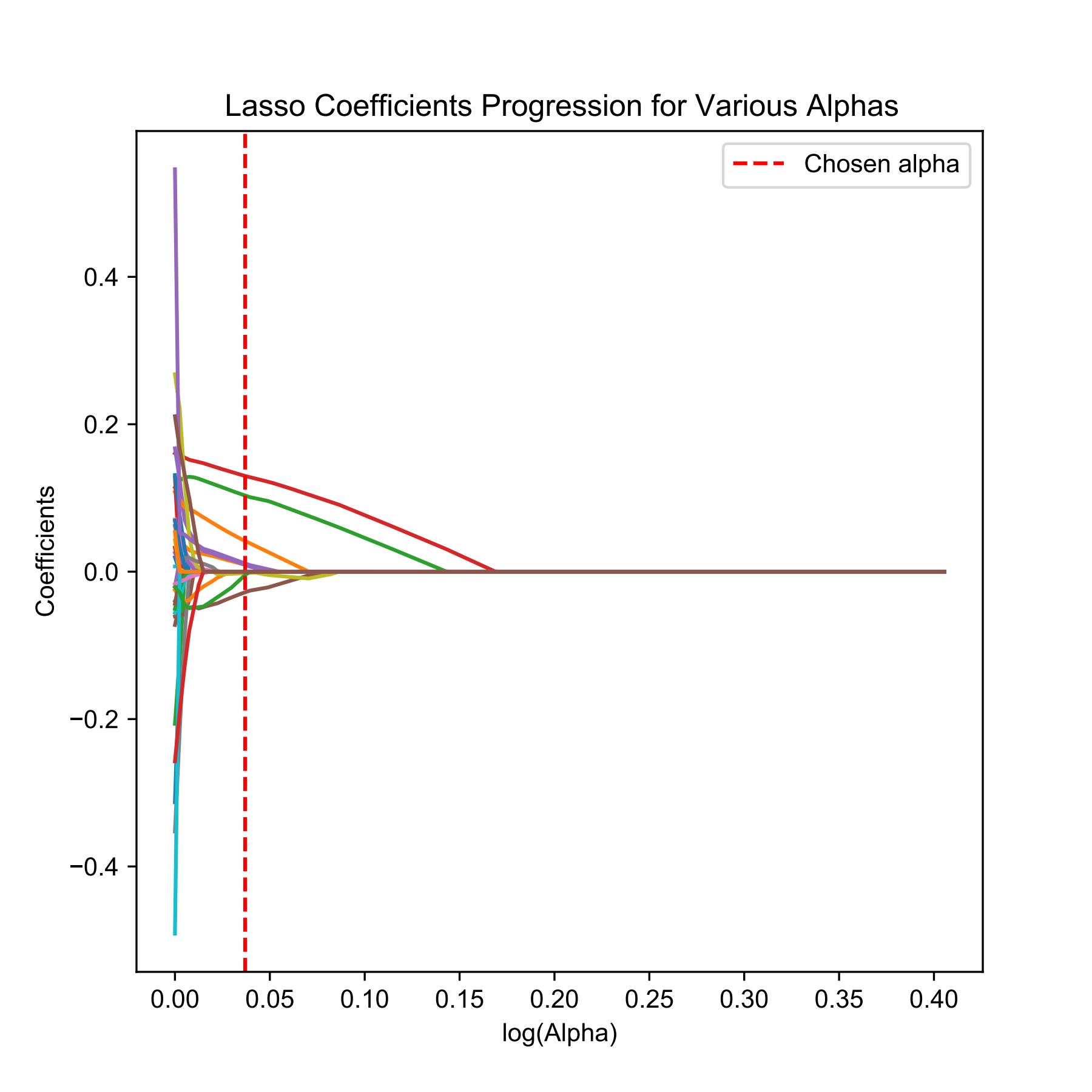

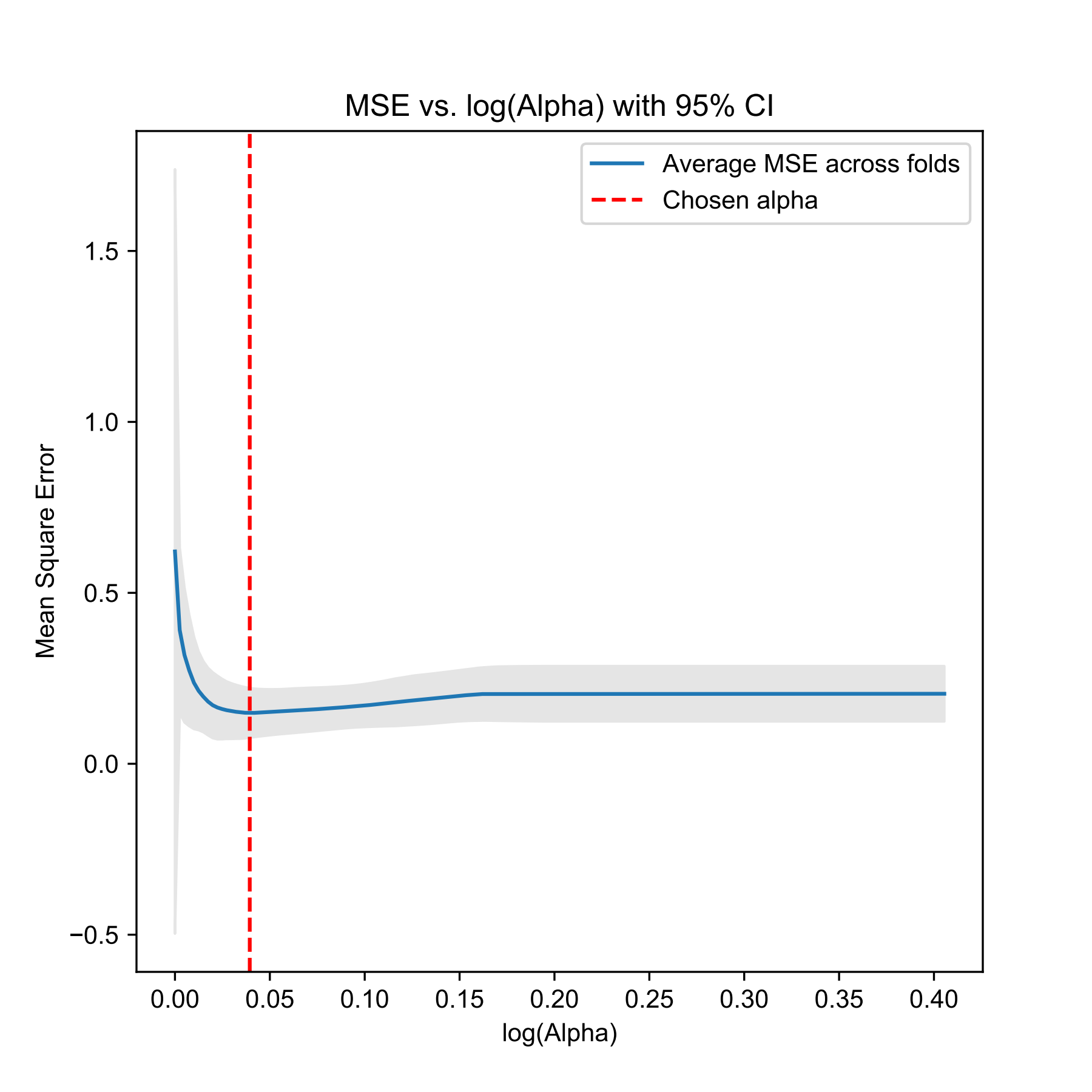

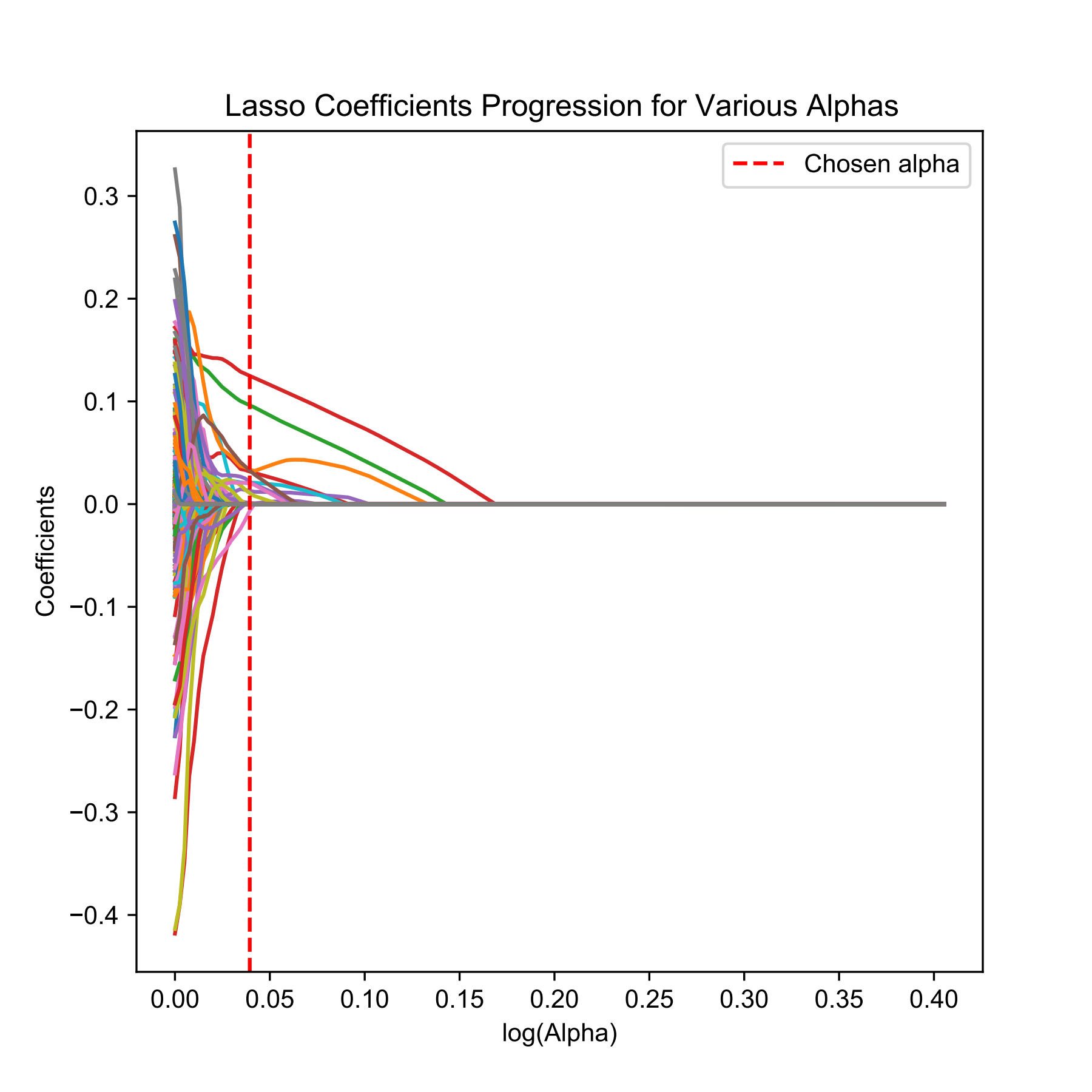

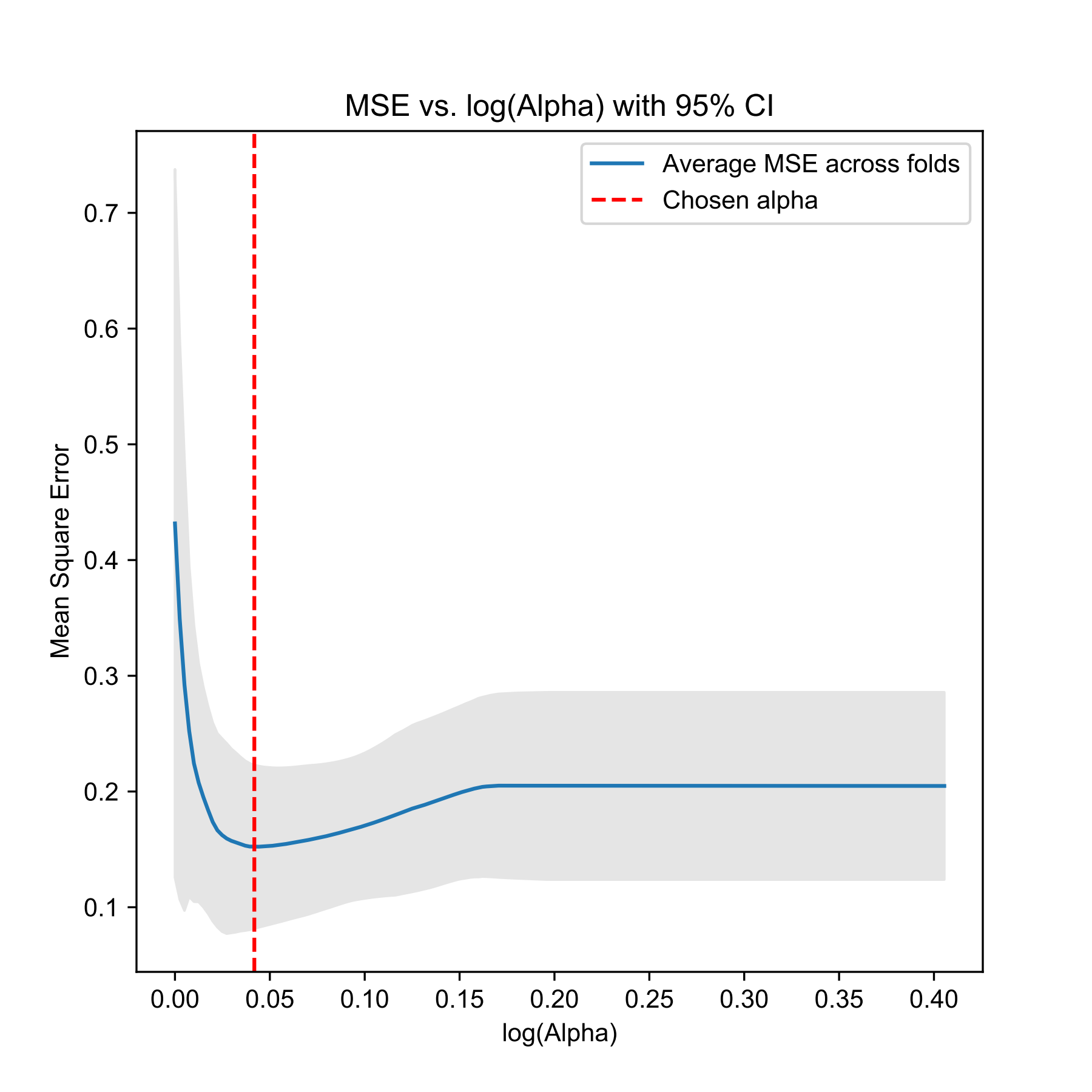

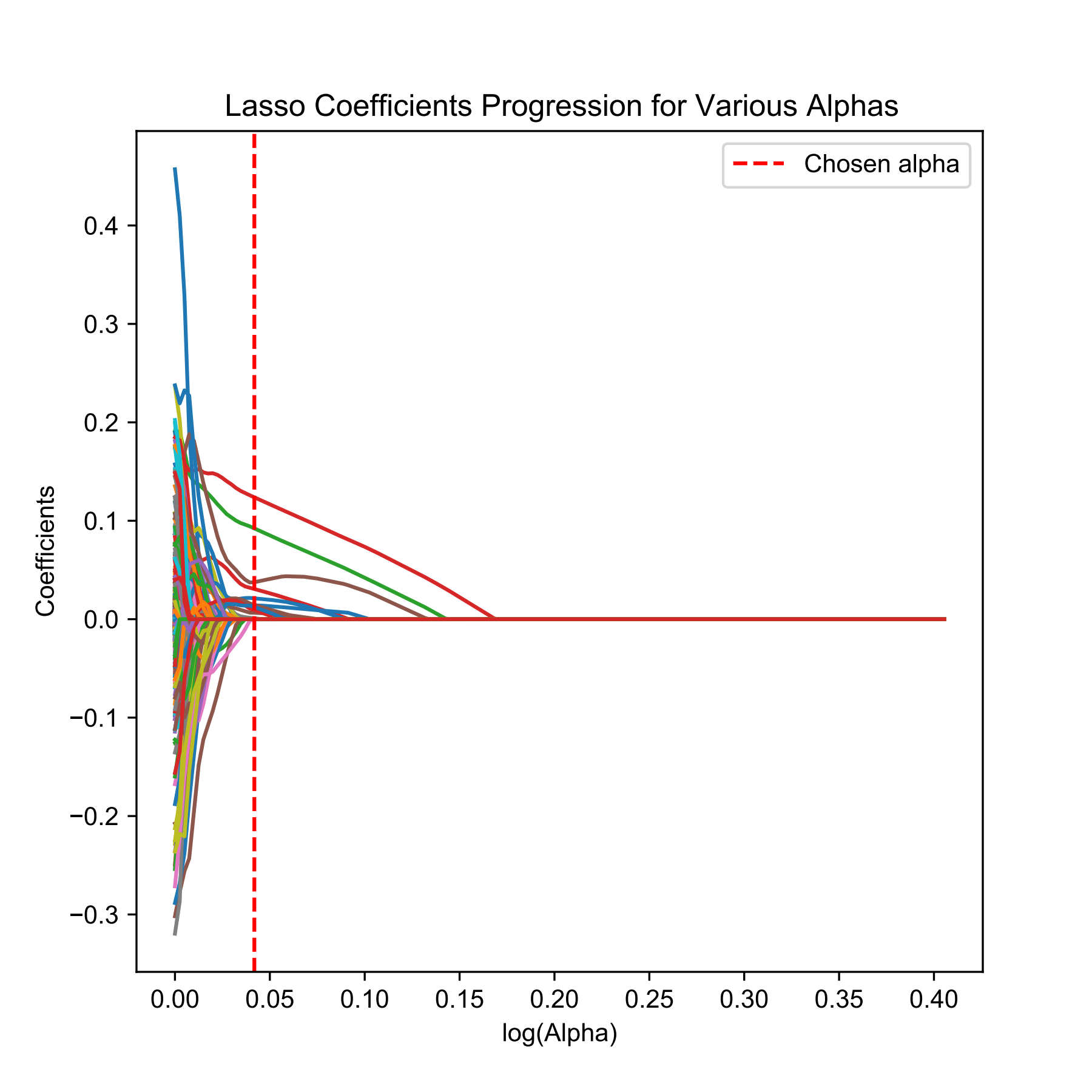


(A)

(B)

(C)

(E)

(D)

(F)

Supplementary Figure 4. Performance of Rad model and DL model. ROC curves in the training(A) and validation(B)set. DCA in in the training(C) and validation(D)set. Calibration curves in the training(E) and validation(F)set.

Supplementary Figure 5. Performance of Clinical model and pre-fusion modes. Calibration curves in the training(A) and validation(B)set. DCA in in the training(C) and validation(D)set.


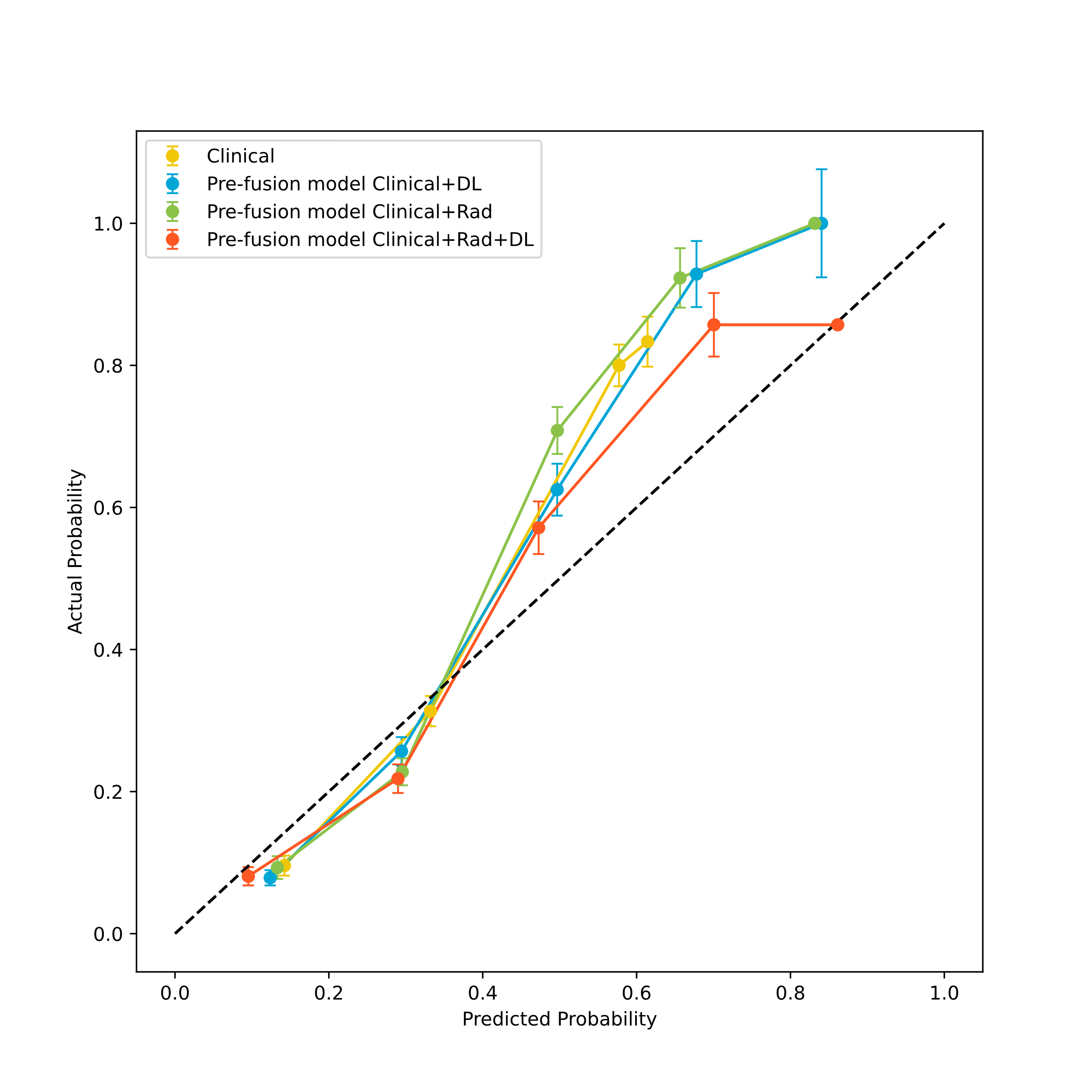

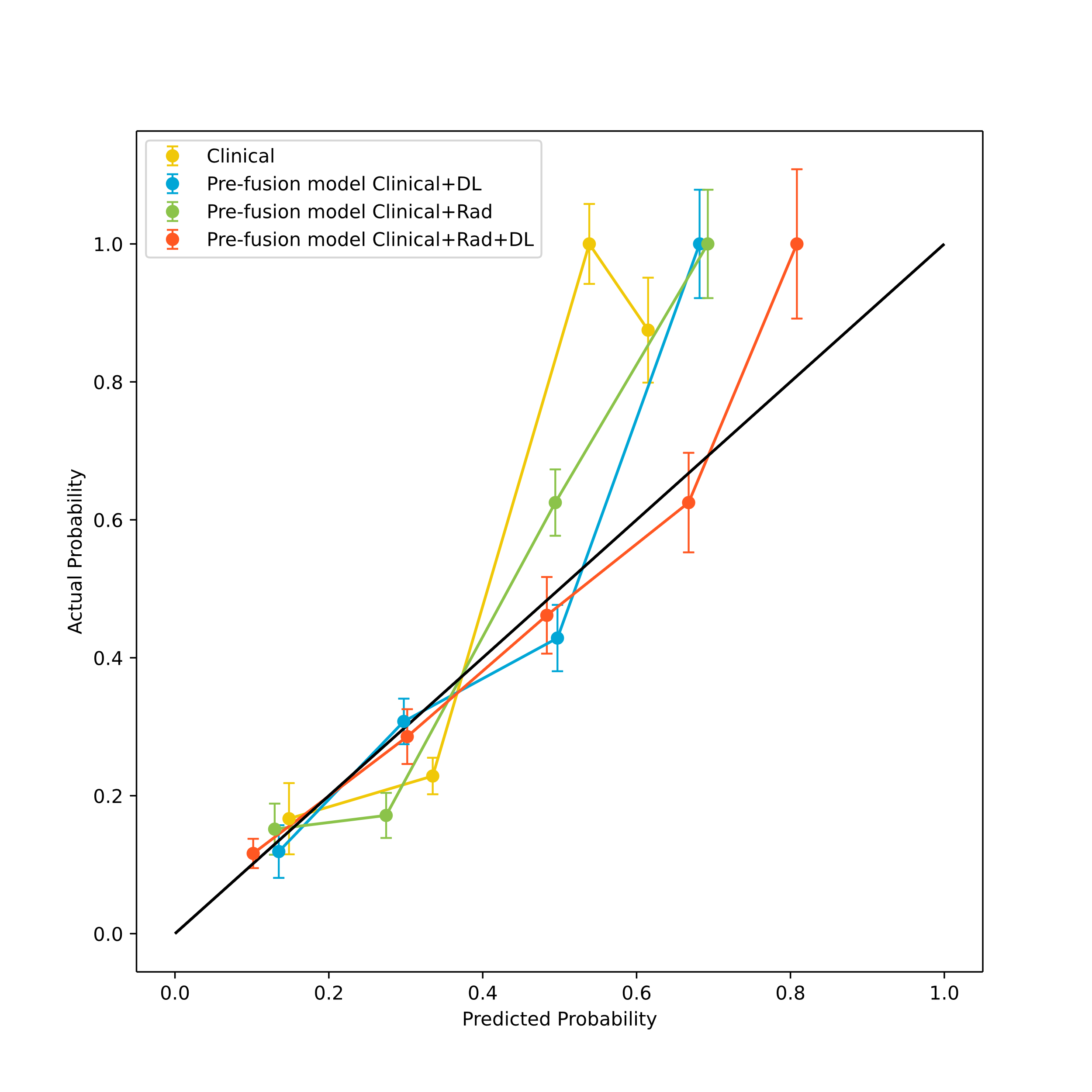

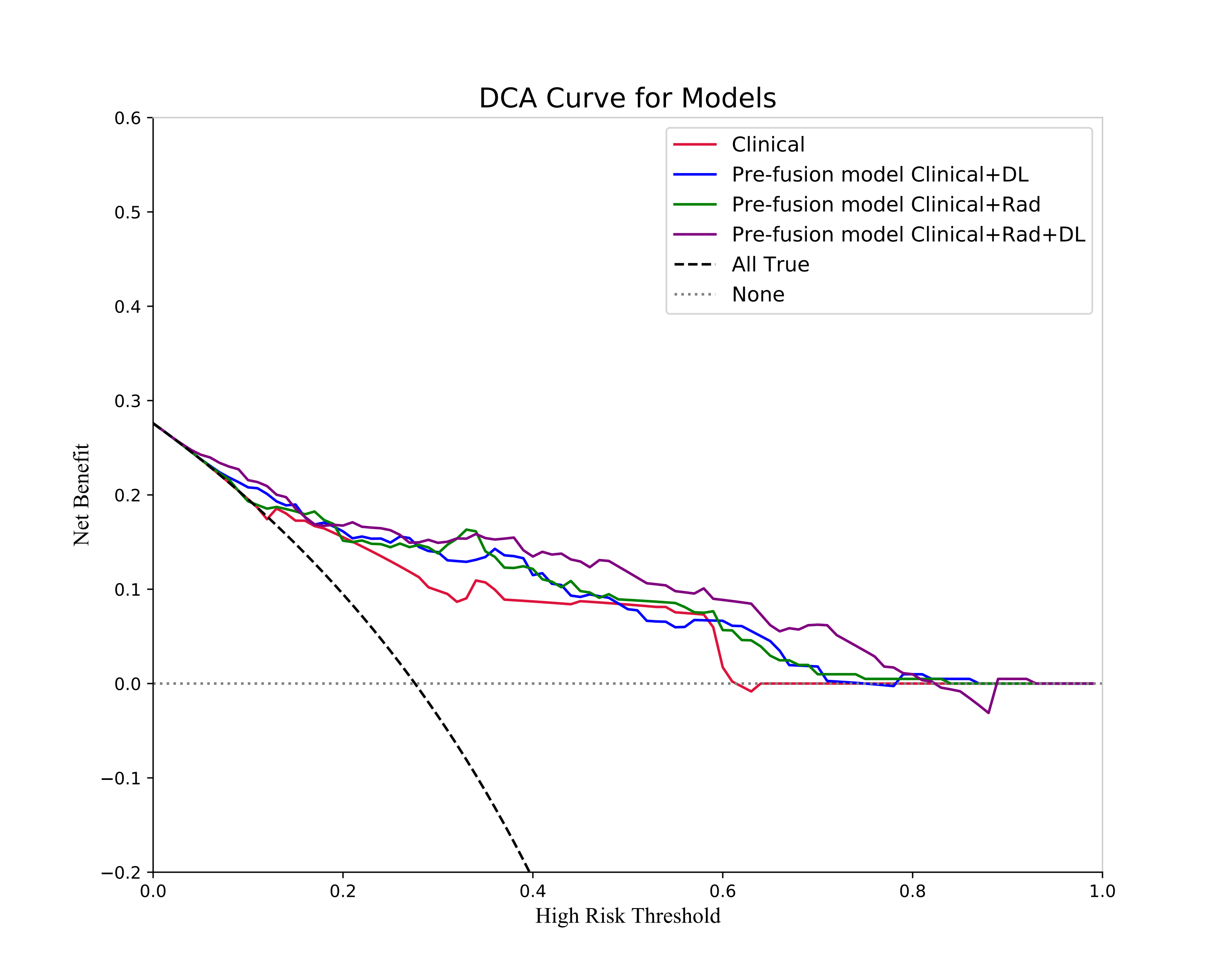

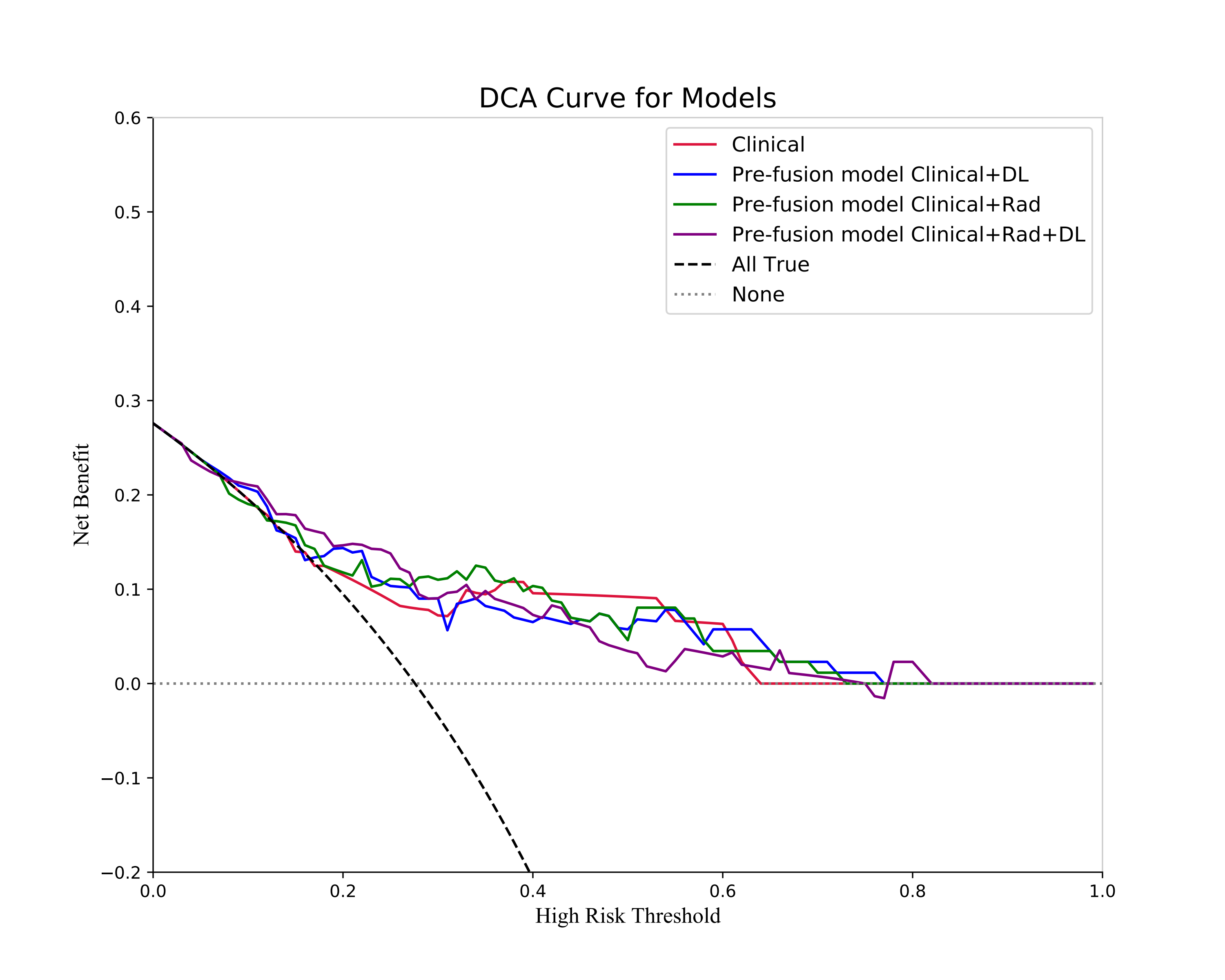


(A)

(B)

(C)

(D)
